# Prospect of Sodium–Glucose Co-transporter 2 Inhibitors Combined With Insulin for the Treatment of Type 2 Diabetes

**DOI:** 10.3389/fendo.2020.00190

**Published:** 2020-04-15

**Authors:** Yinqiu Yang, Chenhe Zhao, Yangli Ye, Mingxiang Yu, Xinhua Qu

**Affiliations:** ^1^Department of Endocrinology, Zhongshan Hospital, Fudan University, Shanghai, China; ^2^Department of Bone and Joint Surgery, Renji Hospital, Shanghai Jiao Tong University School of Medicine, Shanghai, China

**Keywords:** insulin, sodium–glucose co-transporter 2 inhibitor, type 2 diabetes, combination therapy, hyperglycemia

## Abstract

Sodium–glucose co-transporter 2 (SGLT2) inhibitors are a new family of antidiabetic drugs that reduce blood glucose independent of insulin. In this review, we present the advantages and adverse effects of SGLT2 inhibitors plus insulin therapy as a treatment regimen for patients with type 2 diabetes (T2D). Compared with placebo, SGLT2 inhibitors plus insulin therapy could significantly decrease fasting blood glucose and HbA1c, thereby reducing the daily required dose of insulin. A reduction in body weight and improvements in insulin resistance and β-cell function have also been widely reported with this therapy, and other potential advantages, including the reduction in blood pressure, adverse cardiovascular outcomes, and visceral adipose tissue volume, have been revealed. SGLT2 inhibitors cause a greater reduction than dipeptidyl peptidase-4 (DPP-4) inhibitors in body weight and the risk of cardiovascular disease. Furthermore, compared with glucagon-like peptide-1 (GLP-1) agonists, SGLT2 inhibitors reduce blood pressure, and heart failure. As this therapy is an oral preparation, an improvement in patient compliance is also achieved. Despite these advantages, however, combination therapy with SGLT2 inhibitors and insulin has several risks. Although no difference has been found in the incidence of hypoglycemic events and urinary tract infection between the administration of this combination and that of placebo, the risk of genital tract infections was reported to increase with the combination therapy. Additionally, bone adverse effects, euglycemic diabetic ketoacidosis, and volume depletion—and osmotic diuresis—related adverse effects have been observed. Altogether, we could conclude that SGLT2 inhibitors plus insulin therapy is an efficient treatment option for patients with T2D, especially those requiring high daily insulin doses and those with insulin resistance, obesity, and a high risk of cardiovascular events. However, careful monitoring of the adverse effects of this combination is also warranted.

## Introduction

The incidence of type 2 diabetes (T2D) is continuously increasing. Owing to acute complications, such as ketoacidosis and hypertonic coma, or chronic complications, such as nephropathy, vasculopathy, neuropathy, and retinopathy, the health and quality of life of patients with T2D have been severely affected, thereby increasing the health burden. Although numerous oral antidiabetic drugs are available, including sulfonylureas, meglitinides, metformin, thiazolidinediones, glucagon-like peptide-1 (GLP-1) agonists, and dipeptidyl peptidase-4 (DPP-4) inhibitors, maintaining long-term optimal blood glucose control has been difficult in most patients with T2D, even those administered with antidiabetics plus insulin (1–3). Chronic hyperglycemia results in a phenomenon called “glucose toxicity,” which reduces β-cell function and endogenous insulin secretion, ultimately causing impaired insulin sensitivity. This “glucose toxicity” contributes to the progressive worsening of hyperglycemia (4), and as the disease progresses, most patients with T2D require higher doses of insulin to maintain glycemic control (5). Increasing the daily insulin dose might, however, increase insulin resistance, resulting in a high hypoglycemia risk and an increase in body weight.

Sodium–glucose co-transporter 2 (SGLT2) inhibitors are a new family of antidiabetic drugs that reduce blood glucose independent of insulin sensitivity and secretion. As a result, these inhibitors differ from other oral antidiabetic drugs. Currently, there are four types of SGLT2 inhibitors available in Europe, America, and Japan, namely, canagliflozin, dapagliflozin, empagliflozin, and ertugliflozin, the first three of which are also available in China. Three other types of SGLT2 inhibitors, namely, tofogliflozin, ipragliflozin, and luseogliflozin are also available in Japan. Among the different SGLT2 inhibitors, empagliflozin is 2,500-fold more selective for SGLT2 than SGLT1; whereas tofogliflozin, dapagliflozin, ipragliflozin, and canagliflozin are 1,875, 1,200, 550, and 250-fold, respectively, more selective for SGLT2 than SGLT1 (6). Dapagliflozin and ipragliflozin are long-acting SGLT2 inhibitors, whereas canagliflozin, tofogliflozin, luseogliflozin, and empagliflozin are intermediate-acting inhibitors.

The insulin-independent hypoglycemic mechanism indicates that theoretically, SGLT2 inhibitors might be effective in patients with any stage of diabetes and particularly effective in those with severe insulin resistance and receiving high-dosage insulin therapy. Previously, SGLT2 inhibitors plus insulin therapy was reported to improve glycemic control, reduce daily insulin requirements, and mitigate insulin-related weight gain. However, the side effects of SGLT2 inhibitors should be considered.

In this review, we aimed to elucidate the benefits and risks of SGLT2 inhibitors plus insulin therapy on patients with T2D and identify patients that may benefit from the use of SGLT2 inhibitors as a first-line therapy.

## Advantages of SGLT2 Inhibitors Plus Insulin Therapy

### Glycemia-Lowering Effect

#### Reduction in HbA1c and Fasting Blood Glucose Level

In 2017, a meta-analysis of nine randomized controlled trials consisting of a total of 3,069 patients revealed that patients administered with SGLT2 inhibitors plus insulin therapy, compared with the control patients, had a reduction in HbA1c level [*n* = 1,000, MD−1.35%, 95% confidence interval (CI)−2.36 to −0.34; *p* = 0.009]. Additionally, a minor but significant reduction in fasting blood glucose (FBG) level (*n* = 905, MD−1.01 mmol/L, 95% CI−1.98 to 0.04; *p* = 0.04) was observed (7).

A double-blind randomized controlled clinical trial called the Canagliflozin Cardiovascular Assessment Study reported that 100 and 300 mg of canagliflozin plus insulin therapy vs. placebo reduced the level of HbA1c by 0.62% (95% CI 0.54–0.69; *p* < 0.001) vs. −0.73% (95% CI 0.65–0.81; *p* < 0.001) after 18 weeks and 0.58% (95% CI 0.48 to −0.68; *p* < 0.001) vs. 0.73% (95% CI 0.63–0.83; *p* < 0.001) after 52 weeks. Additionally, a significant decrease in the level of FBG was observed after 18 weeks (canagliflozin 100 mg: 1.2 mmol/L, 95% CI 0.9–1.4; 300 mg: 1.6 mmol/L, 95% CI 1.3–1.8) and 52 weeks (canagliflozin 100 mg: 1.1 mmol/L, 95% CI 0.9–1.4; 300 mg: 1.5 mmol/L, 95% CI 1.2–1.7) (8). Clinical studies conducted with other types of SGLT2 inhibitors, such as dapagliflozin and empagliflozin, also reported a reduction in HbA1c and FBG levels (9–13) (Table 1).

**Table 1:** Comparison of the SGLT2 inhibitors and placebo combined with insulin therapy for the treatment of T2D.

**References**	**Type of SGLT2**	**Time (weeks)**		**Change in HbA1c (%)**	**Change in FBG (mg/dl)**	**Change in total daily insulin dose (U)**	**Change in body weight (kg)**	**Change in blood pressure (SBP/DBP mmHg)**
Wilding et al. (13)	Dapagliflozin	12	10 mg	−0.70 (−1.1 to −0.3)	−15.4 (−38.4, 7.5)	−3.1 (−10.7, 4.6) [−5.57%]	−2.6 (−4.0 to −1.2)	−7.2 /−1.2
			20 mg	−0.78 (−1.2 to −0.4)	−27.4 (−50.3 to −4.6)	−2.5 (−10.2 to 5.1) [−4.49%]	−2.4 (−3.8 to −1.0)	−6.1 /−3.9
Wilding et al. (11, 12)	Dapagliflozin	24	2.5 mg	−0.40 (−0.54 to −0.25)	No data	−7.60 (−10.32 to −4.87) [−9.58%]	−1.35 (−1.90 to −0.80)	−0.66 (−3.32 to 2.00) /−0.25 (−1.77 to 1.26)
			5 mg	−0.49 (−0.65 to −0.34)		−6.28 (−8.99 to −3.58) [−7.91%]	−1.42 (−1.97 to −0.88)	−2.37 (−5.01 to 0.26) /−1.18 (−2.68 to 0.32)
			10 mg	−0.57 (−0.72 to −0.42)		−6.82 (−9.56 to −4.09) [−8.59%]	−2.04 (−2.59 to −1.48)	−3.11 (−5.79 to −0.43) /−0.84 (−2.36 to 0.69)
		48	2.5 mg	−0.32 (−0.48 to −0.16)	−0.54 (−1.05 to −0.04)	−11.4 (−15.5 to −7.4) [−13.53%]	−1.78 (−2.53 to −1.03)	−3.81 (−6.65 to −0.97) /−1.65 (−3.30 to −0.00)
			5/10 mg	−0.49 (−0.65 to −0.33)	−0.68 (−1.18 to −0.17)	−10.2 (−14.3 to −6.2) [−12.11%]	−1.82 (−2.56 to −1.07)	−2.84 (−5.67 to −0.01) /−1.33 (−2.98 to 0.31)
			10 mg	−0.53 (−0.70 to −0.37)	−0.92 (−1.43 to −0.41)	−11.2 (−15.3 to −7.2) [−13.30%]	−2.43 (−3.18 to −1.68)	−2.61 (−5.48 to 0.27) /−1.54 (−3.20 to 0.12)
		104	2.5 mg	−0.21 (−0.41 to −0.01)	−0.14 (−0.73 to 0.45)	−14.3 (−20.5 to −8.0) [−15.49%]	−2.81 (−3.87 to −1.75)	No data
			5/10 mg	−0.39 (−0.59 to −0.18)	−0.89 (−1.48 to −0.31)	−16.8 (−23.1 to −10.5) [−18.20%]	−2.86 (−3.92 to −1.80)	−2.6/−2.9
			10 mg	−0.35 (−0.55 to −0.15)	−0.31 (−0.89 to 0.28)	−19.2 (−25.5 to −12.9) [−20.80%]	−3.33 (−4.38 to −2.27)	−7.5/−4.0
Rosenstock (10)	Empagliflozin	18	10 mg	−0.44 (−0.59 to −0.29)	−1.17 (−1.62 to −0.71)	No data	−1.31 (−1.82 to −0.80)	−2.4 (−4.7 to −0.2) /−1.0 (−2.4 to 0.4)
			25 mg	−0.52 (−0.67 to −0.37)	−1.55 (−2.00 to −1.09)		−1.88 (−2.39 to −1.37)	−1.7 (−3.9 to 0.6) /−0.7 (−2.1 to 0.7)
		52	10 mg	−0.38 (−0.59 to −0.16)	−0.69 (−1.23 to −0.15)	−8.8 (−14.8 to −2.8) [−8.70%]	−2.39 (−3.40 to −1.39)	−0.6 (−3.4 to 2.3) /−0.7 (−2.4 to 1.1)
			25 mg	−0.46 (−0.67 to −0.25)	−0.79 (−1.33 to −0.26)	−11.2 (−17.2 to −5.2) [−11.07%]	−2.48 (−3.48 to −1.47)	−0.9 (−3.7 to 1.9) /−1.9 (−3.7 to −0.1)
Rosenstock and Ferrannini (14)	Empagliflozin	18	10 mg	−0.6 (−0.8 to −0.4)	−1.6 (−2.1 to −1.1)	No data	−1.7 (−3.3 to −0.1)	−3.4 (−6.0 to −0.8) /−3.3 (−5.1 to −1.5)
			25 mg	−0.7 (−0.9 to −0.5)	−1.6 (−2.1 to −1.1)		−0.9 (−2.5 to 0.8)	−3.0 (−5.7 to −0.4) /−1.7 (−3.5 to 0.1)
		78	10 mg	−0.5 (−0.7 to −0.2)	−0.7 (−1.2 to −0.2)	−6.7 (−10.9 to −2.4) [−12.74%]	−2.9 (−4.3 to −1.5)	−4.2 (−7.0 to −1.3) /−2.6 (−4.5 to −0.8)
			25 mg	−0.6 (−0.9 to −0.4)	−1.0 (−1.5 to −0.5)	−5.9 (−10.4 to −1.5) [−11.22%]	−2.8 (−4.2 to −1.3)	−2.4 (−5.4 to 0.5) /−1.3 (−3.1 to 0.6)
Neal et al. (8)	Canagliflozin	18	100 mg	−0.62 (−0.69 to −0.54)	−1.2 (−1.4 to −0.9)	No data	−1.9 (−2.2 to −1.6)	−2.3 (−3.7 to −1.0) /−1.0 (−1.8 to −0.2)
			300 mg	−0.73 (−0.81 to −0.65)	−1.6 (−1.8 to −1.3)		−2.4 (−2.7 to −2.1)	−4.1 (−5.5 to −2.8) /−1.7 (−2.5 to −0.9)
		52	100 mg	−0.58 (−0.68 to −0.48)	−1.1 (−1.4 to −0.9)	−2 [−3.33%]	−2.8 (−3.3 to −2.4)	−3.1 (−4.6 to −1.7) /−1.2 (−2.0 to −0.3)
			300 mg	−0.73 (−0.83 to −0.63)	−1.5 (−1.7 to −1.2)	−4.3 [−7.17%]	−3.5 (−3.9 to −3.0)	−6.2 (−7.7 to −4.8) /−2.4 (−3.2 to −1.5)

*SGLT2, sodium–glucose co-transporter 2; T2D, type 2 diabetes; FBG, fasting blood glucose; SBP, systolic blood pressure; DBP, diastolic blood pressure*.

Recent head-to-head studies reported that GLP-1 agonists cause superior reduction in the level of HbA1c relative to SGLT2 inhibitors (15–17). However, most of the currently marketed GLP-1 agonists are injection preparations, which may affect patient compliance. By comparing SGLT2 inhibitors to DPP-4 inhibitors combined with insulin therapy for patients with T2D, a meta-analysis revealed that the former resulted in better glycemic control and lower levels of HbA1c [weighted mean difference (WMD) 0.24%, 95% CI 0.05–0.43] and FBG (WMD 18.0 mg/dl, 95% CI 7.6–28.5 mg/dl) than the latter (18). However, no significant difference was observed between SGLT2 inhibitors and pioglitazone plus insulin as treatment regimens for patients with T2D (19).

#### Reduction in Daily Insulin Dose

A 2017 meta-analysis reviewed nine randomized controlled trials that compared SGLT2 inhibitors plus insulin therapy with placebo plus insulin therapy. Trials that administered multiple daily subcutaneous injections of basal plus bolus insulin therapy and insulin pump were included. Based on the findings, SGLT2 inhibitors could decrease the total daily dosage of insulin (*n* = 813, MD 4.85 U/24 h, 95% CI 2.29–7.42, *p* = 0.002) in the placebo-controlled trials (7).

Several randomized controlled clinical trials have reported a reduction in total daily insulin dose (i.e., basal plus bolus insulin dose or the total daily dose of continuous insulin infusion pump) after a group administered with SGLT2 inhibitors plus insulin was compared with a group administered with placebo plus insulin (8–13) (Table 1). Harris et al. found that for patients prescribed a high insulin regimen (100 IU/day), SGLT2 inhibitors could cause a more significant reduction in the insulin dose. For patients receiving 101–200 IU of insulin per day, canagliflozin caused a 17-IU reduction in insulin dose at 3 and 6 months. Moreover, for those receiving 200 IU of insulin daily, insulin dose reductions of 21 and 23 IU, and 77 and 71 IU were observed at 3 and 6 months when canagliflozin and dapagliflozin were, respectively, administered (20).

According to a meta-analysis by Min et al. combining insulin therapy with SGLT2 and DPP-4 inhibitors results in a greater reduction in the total daily insulin dose than combining insulin therapy with placebo (WMD 6.40 IU/day, 95% CI 3.82–8.97 IU/day, *p* < 0.001 and WMD 1.86 IU/day, 95% CI 0.45–3.27 IU/day, *p* = 0.010, respectively). However, the differences in the insulin-reducing effects between the SGLT2 and DPP-4 inhibitors were not statistically significant (18). In a comparative review by Singh and Singh, DPP-4 inhibitors plus insulin resulted in a 10–20% reduction in total daily insulin dose, whereas SGLT2 inhibitors plus insulin only resulted in a 0–10% reduction (21). A study comparing SGLT2 inhibitors with pioglitazone reported that combining both drugs with insulin therapy significantly decreased the insulin demand relative to the placebo; however, the difference in total daily insulin dose reduction between the two groups was not significant (19).

### Reduction in Body Weight

Insulin therapy often results in an increase in body weight (22, 23). Combining insulin with other antidiabetic drugs, such as sulfonylurea and thiazolidinedione, also results in significant weight gain (24–26).

Several clinical trials (8–13) and a meta-analysis (7) have reported that combining SGLT2 inhibitors with insulin as a treatment regimen for T2D would impede this weight gain and would result in a significant decrease in body weight (Table 1). Compared with other antidiabetic drugs, such as pioglitazone and DPP-4 inhibitors, SGLT2 inhibitors can significantly accelerate weight reduction (18, 19, 21). However, compared with GLP-1 agonists, the results are controversial. A network meta-analysis and a head-to-head clinical trial (SUSTAIN-8) reported better body weight reduction with SGLT2 inhibitors, whereas two additional head-to-head clinical trials (DURATION-8 and PIONEER-2) reached different conclusions (15–17, 27).

Animal studies have also demonstrated the achievement of weight loss owing to treatment with SGLT2 inhibitors. After 8 weeks of tofogliflozin intake, high fat diet (HFD)-induced hepatic steatosis and weight gain were inhibited in rats (28). After the administration of dapagliflozin for 35 days, body weight was significantly reduced, and lipid decomposition was enhanced (29). Consistent with animals administered with other SGLT2 inhibitors, HFD-induced obese mice administered with empagliflozin had a reduction in fatty liver and weight gain (30).

### Improvement in Insulin Resistance and β-Cell Function

DeFronzo et al. employed a glucose clamp test to measure insulin resistance in patients with T2D after 2 weeks of treatment with SGLT2 inhibitors. Compared with the placebo group, muscle glucose uptake was significantly increased in the SGLT2 inhibitor group, indicating an improvement in peripheral insulin resistance (31). Ferrannini et al. reported that a single dosage of empagliflozin enhanced glycosuria and resulted in improved insulin sensitivity and β-cell function for tissue glucose uptake (32).

A study using a rat model of T2D with pancreatectomy found that chronic glycosuria induced by phlorizin improved insulin resistance and restored β-cell function (33). In SGLT2 knockout mice, glucose tolerance and β-cell function were demonstrated (34). By conducting the hyperinsulinemic–euglycemic clamp test in mice treated with tofogliflozin for 8 weeks, significant improvements in glucose infusion rate (GIR) and systemic insulin resistance were observed. Additionally, muscle glucose uptake was increased, suggesting that fat storage was reduced and peripheral insulin resistance was improved (28). When the glucose and insulin tolerance tests were performed with HFD-induced obese mice, empagliflozin was observed to improve glucose intolerance and insulin resistance in the fasting and fed states (30).

### Cardiovascular Benefits

The Empagliflozin Cardiovascular Outcome Event Trial, which consisted of 7,020 T2D patients, found that routine treatment with empagliflozin significantly reduced cardiovascular mortality by 38% [hazard ratio (HR) 0.62, 95% CI 0.49–0.77, *p* < 0.001] and hospitalization due to heart failure by 35% (HR 0.65, 95% CI 0.50–0.85, *p* = 0.002). In this trial, ~48% of patients were administered with insulin. The HR for primary composite outcome in the subgroup of insulin users was 0.93 (95% CI 0.75–1.13) (35). Several studies have also reported a significant decrease in blood pressure owing to SGLT2 inhibitor plus insulin treatment (7–13) (Table 1). A 2016 meta-analysis of six regulatory submissions and 57 published clinical trials with more than 70,000 participants sought to analyze seven different SGLT2 inhibitors. Based on the findings, SGLT2 inhibitors exhibit protective effects against cardiovascular diseases and death. In fact, by using SGLT2 inhibitors, the risk of major adverse cardiovascular events was significantly decreased [relative risk (RR) 0.84, 95% CI 0.75–0.95, *p* = 0.006], similar to cardiovascular death (RR 0.63, 95% CI 0.51–0.77, *p* < 0.0001) and heart failure (RR 0.65, 95% CI 0.50–0.85, *p* = 0.002). No statistically significant difference in cardiovascular outcomes was found between the different SGLT2 inhibitors (36). Clinical trials on other SGLT2 inhibitors, such as canagliflozin, dapagliflozin, and ertugliflozin, and their cardiovascular outcomes in T2D patients are still ongoing (6).

GLP-1 agonists have been reported to reduce the risk of cardiovascular disease; however, SGLT2 inhibitors cause a relatively significant reduction in systolic blood pressure and play a unique role in the prevention of heart failure relative to GLP-1 agonists (37). Previously, a multinational observational analysis was performed with patients prescribed antidiabetic drugs during 2012 and 2015 in Sweden, Norway, and Denmark. By using the propensity score to match SGLT2 inhibitor users (*n* = 22,830) to users of other hypoglycemic drugs (*n* = 68,490), the study found that compared with other hypoglycemic drugs, the SGLT2 inhibitors decreased major adverse cardiovascular events (HR 0.78, 95% CI 0.69–0.87, *p* < 0.0001), cardiovascular disease mortality (HR 0.53, 95% CI 0.40–0.71, *p* < 0.0001), and hospital events due to heart failure (HR 0.70, 95% CI 0.61–0.81, *p* < 0.0001) (38). A comparative review sought to compare DPP-4 inhibitors and SGLT2 inhibitors as add-on therapies with insulin and report the cardiovascular benefits of empagliflozin. According to the review, SGLT2 inhibitors plus insulin therapy is an ideal combination for T2D patients without renal dysfunction (21).

None of the current studies sought to directly compare combination therapy with SGLT2 inhibitors and insulin with monotherapy with SGLT2 inhibitors on cardiovascular outcomes. Therefore, whether SGLT2 inhibitors plus insulin combination therapy could affect the notable improvement in cardiovascular outcomes achieved with SGLT2 inhibitor monotherapy remains unclear. Nonetheless, SGLT2 inhibitors plus insulin therapy has definitive cardiovascular benefits compared with those of monotherapy with insulin or combination therapy with insulin and other drugs.

### Other Potential Advantages

In the trials performed with dapagliflozin and canagliflozin combined with insulin, the albumin-to-creatinine ratio was significantly improved (8, 12). Canagliflozin has been reported to reduce triglycerides and albuminuria (8), whereas dapagliflozin has been reported to decrease liver proton-density fat fraction, liver volume, visceral adipose tissue volume, interleukin-6, and N-terminal pro-B-type natriuretic peptide (18). A further study should, however, be conducted to confirm these findings.

## Mechanisms Underlying the Advantages of SGLT2 Inhibitors Plus Insulin Therapy

### Mechanisms Underlying the Glycemia-Lowering Effect

The kidneys play a key role in glucose metabolism. After glucose filtration by the glomerulus, glucose is reabsorbed in the proximal tubule. Thereafter, it is transported through the phospholipid bilayer of the cell membrane via glucose transporters. SGLTs are a class of transporters found in the small intestinal mucosa and proximal convoluted tubules of the kidney. Of these transporters, SGLT2 is predominant (39). SGLT2 is mainly expressed in the luminal membranes of renal proximal convoluted tubules, with high transport capacity and low affinity, enabling 90% of glucose reabsorption from the glomerular filtrate in segments S1 and S2. The remaining 10% of glucose reabsorption is completed in segment S3 by SGLT1 (6, 40).

In patients with T2D, the expression level of SGLT2 is enhanced in renal cells (41). Blocking SGLT2 could thus decrease the renal threshold for glucose (RTG), leading to osmotic diuresis (6). Although 90% of glucose reabsorption is carried out by SGLT2, SGLT2 inhibitors only reduce glucose absorption by 30–50% in patients with diabetes. This is because of residual SGLT2 activity and SGLT1 compensation (42, 43).

### Mechanisms Underlying Weight Loss

**Figure 1:**
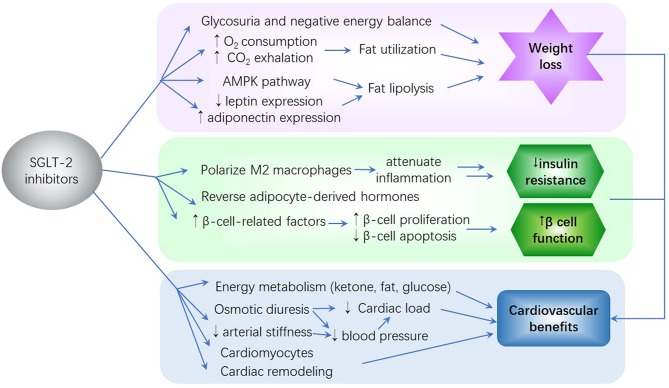
The potential mechanism of sodium–glucose co-transporter 2 (SGLT2) inhibitors on glycemia reduction, weight reduction, insulin resistance, β-cell function improvement, and reduction of cardiovascular complications. (1) SGLT2 inhibitors cause glycosuria and negative energy balance, thereby leading to body weight loss. (2) SGLT2 inhibitors improve insulin resistance and β-cell function by attenuating inflammation, affecting adipocyte-derived hormones, and promoting β-cell-related factor expression. (3) SGLT2 inhibitors improve energy utilization, cardiac efficiency, and contractility. These inhibitors reduce cardiac load and blood pressure. The effects of SGLT2 inhibitors on cardiomyocytes and cardiac remodeling result in improved cardiac function. Moreover, their ability to mitigate insulin resistance, glucose variability, visceral adiposity, oxidative stress, and inflammation and their improvement of kidney function contribute to a reduction in the risk of cardiovascular disease.

Body weight loss is not only due to the calorie loss induced by glycosuria and negative energy balance (28) but also due to other factors (44). First, SGLT2 inhibitors shift the energy metabolism toward fat and sugar utilization. Studies have demonstrated that the increased oxygen consumption and carbon dioxide exhalation caused by SGLT2 inhibitors led to a fuel shift to fat utilization. Furthermore, tissue glucose treatment is decreased and lipid use is increased (30, 45). SGLT2 inhibitors increase the β-oxidation of fatty acids by activating the adenosine monophosphate-activated protein kinase (AMPK) pathway and changing adiponectin and leptin expression. Hawley et al. found that SGLT2 inhibitors activate AMPK and reduce liver lipid content, promoting fatty acid oxidation (46). In the skeletal muscle of HFD-induced obese mice, empagliflozin was found to increase AMPK and acetyl-CoA carboxylase phosphorylation (30), indicating that SGLT2 inhibitors enhance the β-oxidation of free fatty acids by activating the AMPK pathway. SGLT2 inhibitors also regulate adiponectin and leptin expression in white adipose tissue and increase the oxidation of fatty acids. Leptin and adiponectin, which are known as adipose tissue-specific adipokines, are essential in the regulation of food intake and energy homeostasis (47). Empagliflozin administration downregulates leptin expression and upregulates adiponectin mRNA expression. Serum leptin level increases with body weight gain, promoting the esterification of fatty acids to triglycerides and increasing hydrolysis, thereby leading to a net flow of fatty acids out of the cell. Conversely, adiponectin increases the β-oxidation of fatty acids and decreases the serum level of triglycerides and free fatty acids. Therefore, the increase in adiponectin expression and decrease in leptin expression contribute to lipolysis and energy expenditure, thereby leading to a decrease in body weight (44) (Figure 1).

### Mechanisms Underlying Improved Insulin Resistance and β-Cell Function

Current studies have analyzed the potential mechanisms whereby SGLT2 inhibitors improve insulin resistance and β-cell function in patients with T2D (48) (Figure 1).

#### SGLT2 Inhibitors Attenuate Inflammation and Insulin Resistance by Polarizing M2 Macrophages

Long-term mild inflammation is correlated with obesity, insulin resistance, and T2D (49, 50). Obesity and heterotopic fat accumulation induce innate immune responses and the recruitment of immune cells, promote the infiltration of pro-inflammatory immune cells into metabolic tissues, and cause phenotypic changes in macrophages, ultimately inducing insulin resistance (51, 52). In particular, the recruitment and polarization of macrophages are key to the inflammation and insulin resistance induced by obesity. The inhibition of macrophage M1 polarization and activation of the alternative macrophage, M2, could prevent the aggravation of inflammation and insulin resistance (53–55). Empagliflozin has been reported to reduce macrophage accumulation and lead to M2-dominant phenotypic metastasis of white adipose tissue and liver macrophages, ultimately attenuating obesity-related inflammation (30). Moreover, before the recruitment of M1-polarized macrophages, Th1 and CD8^+^ T cells infiltrate the tissue and interact with macrophages to form an incommensurate feedforward loop, causing fat inflammation and insulin resistance (47, 49). Empagliflozin might also alleviate inflammation and insulin resistance in obesity by decreasing M1 macrophages and T cell accumulation and increasing the number of M2 macrophages.

#### SGLT2 Inhibitors Affect Adipocyte-Derived Hormones

In patients with T2D, serum leptin (56) and FGF-21 (57) levels are increased while serum adiponectin level is decreased (58); these changes are considered to be closely correlated with the occurrence and development of insulin resistance (59, 60). Tahara et al. found that levels of these adipocyte-derived hormones vary in mice and humans with T2D, but these changes could be reversed after the condition of diabetes is improved. Such findings imply that SGLT2 inhibitors reduce insulin resistance in T2D (61). Following the administration of long-acting SGLT2 inhibitors, the Matsuda–DeFronzo index and the disposition index, which are known as parameters of insulin secretion and insulin resistance, respectively, were also improved, indicating that the SGLT2 inhibitors improve insulin resistance and secretion (61). Compared with intermediate-acting SGLT2 inhibitors, the long-acting inhibitors can reduce daily blood glucose excursion, protect pancreatic function, and improve glucose tolerance and insulin resistance (61).

#### SGLT2 Inhibitors Promote the Expression of Different β-Cell-Related Factors

Owing to increased cell proliferation and decreased β-cell apoptosis, a larger pancreatic β-cell mass was observed in mice treated with luseogliflozin. Additionally, the β-cell-related factors were significantly increased in the luseogliflozin-treated mice, and the insulin gene transcription factors, MafA and PDX1, and insulin levels were increased (62). In empagliflozin-treated mice, the expression levels of β-cell-related factors, including insulin, MafA, PDX1, GLP-1 receptor, and glucose transporter Glut2, were increased. Following 1 week of treatment with empagliflozin, β-cell proliferation was promoted, and its protective effects against pancreatic β-cells were observed. However, lipid metabolism was not affected (63). Together, the above results suggest that SGLT2 inhibitors, independent of lipid metabolism, are beneficial for protecting pancreatic β-cell function against glucose toxicity.

### Mechanisms Underlying the Cardiovascular Benefits

Currently, there is no evidence of a synergistic or antagonistic effect between SGLT2 inhibitors and insulin on the cardiovascular system. Further studies are thus needed to clarify the mechanisms underlying their combination effects. However, as an add-on therapy to insulin, SGLT2 inhibitors result in significant cardiovascular benefits. Here, we mainly discussed the mechanisms employed by SGLT2 inhibitors in the cardiovascular system.

First, the protective effects of SGLT2 inhibitors against cardiovascular diseases and death may be partly explained by their effects on energy metabolism. SGLT2 inhibition exerts direct effects on pancreatic α-cells, increasing glucagon concentrations and decreasing insulin level, which ultimately promote ketone production (64). Ketones are a more efficient substrate than glucose in myocardial energy generation following internalization by the heart (65). This shift in fuel metabolism from fat/glucose oxidation to ketone bodies improves energy utilization, thereby contributing to improvements in cardiac efficiency, contractility, and cardiovascular protection (65). SGLT2 inhibitors also mitigate insulin resistance and glucose variability, thereby increasing the risk of atherosclerosis. Furthermore, the reduction in body weight, fat mass, and visceral adiposity is related to the decreased risk of cardiovascular complications.

Second, the effects of SGLT2 inhibitors on renal function may contribute to its cardiovascular benefits (66). Osmotic diuresis and the increasing natriuresis caused by SGLT2 inhibitors at the proximal tubule resulted in a decrease in plasma volume and blood pressure (67). Cherney et al. reported a decrease in arterial stiffness after empagliflozin treatment, which also contributes to the reduction in blood pressure (68). The contraction of the circulating volume could reduce cardiac preload, thereby lowering the ventricular filling pressure. Further, blood pressure reduction could reduce cardiac afterload, which could improve heart function. The preservation of the glomerular filtration rate might also result in some cardiovascular benefits through the avoidance of volume overload and diuretic resistance (69). During chronic renal insufficiency, SGLT2 inhibitors increase sodium delivery to the macula densa, thereby causing the activation of tubule-glomerular feedback, afferent vasoconstriction, and glomerular pressure reduction (70). The effects of SGLT2 inhibitors on kidney function, including proteinuria, and uric acid reduction, might also serve as potential cardiovascular benefits (71).

Third, SGLT2 inhibitors exhibit positive effects on myocardia. High concentrations of sodium and calcium in cardiomyocytes are early markers and drivers of cardiovascular death and heart failure (72). Previously, SGLT2 inhibition was demonstrated to inhibit myocardial Na+/H+ exchanger, reducing myocardial intracellular sodium, leading to a subsequent decrease in intracellular calcium concentrations and an increase in mitochondrial calcium. Regulating mitochondrial calcium concentrations can lower the risk of heart failure and sudden cardiac death (73). Additionally, SGLT2 inhibition may decrease myocardial oxygen demand and increase oxygen delivery to the heart by increasing hematocrit, ultimately resulting in improvements in cardiac function (74).

Finally, SGLT2 inhibitors play a role in cardiac remodeling. After 3 months of treatment with empagliflozin, improvement in diastolic function and a reduction in the left ventricular mass index were observed in patients with T2D (75). In experimental diabetic mouse models, SGLT2 inhibition was found to reduce the expression of prohypertrophic and profibrotic proteins, thereby reversing left ventricular hypertrophy and reducing myocardial fibrosis, which ultimately improved the systolic and diastolic functions of the heart (76, 77).

Other potential mechanisms could also exist. SGLT2 inhibitors can reduce oxidative stress and inflammation, which play a role in the initiation and progression of atherosclerosis (78). Additionally, they can attenuate the pro-thrombotic milieu associated with hyperglycemia through neutrophil-derived S100 calcium-binding proteins A8/A9 (79).

## Risks of SGLT2 Inhibitors Plus Insulin Therapy

### Hypoglycemia

In a prior meta-analysis, there was no difference in hypoglycemia risk between treatment with SGLT2 inhibitors plus insulin therapy and that with placebo (OR 1.18, 95% CI 0.86–1.61, *p* = 0.30) (7). However, SGLT2 inhibitors can significantly reduce the daily administered with a dose of insulin. Blood glucose monitoring is essential at the beginning of SGLT2 inhibitors plus insulin therapy to reduce the hypoglycemic events. Additionally, the dosage of insulin injection should be reduced as appropriate. On the long term, a reduction in daily insulin dose might lower hypoglycemia risk; however, this should be further validated.

### Genital and Urinary Tract Infections

Genital tract infection (GTI) and urinary tract infection (UTI) are the major adverse effects of SGLT2 inhibitors. A meta-analysis reported that UTI and GTI were more common with the administration of SGLT2 inhibitors than with placebo (OR 1.42, CI 1.06–1.90 and OR 5.06, CI 3.44–7.45, respectively) (67). In a meta-analysis that compared SGLT2 inhibitors plus insulin with the control treatment, a significant difference in UTI risk was not found (OR 1.34, 95% CI 0.79–2.27, *p* = 0.28); however, the risk of GTI was higher in the former group (OR 2.96, 95% CI 1.05–8.37, *p* = 0.04) than in the latter group (7). Nonetheless, these infections were mild and displayed a good response to therapy (7, 80).

### Diabetic Ketoacidosis

Many studies have shown that the risk of ketoacidosis might increase in patients administered with SGLT2 inhibitors (81, 82), a pre-existing warning reported by the Food and Drug Administration (FDA) in 2015. Euglycemic diabetic ketoacidosis might be due to a low insulin-to-glucose ratio. SGLT2 inhibitors were found to cause a significant reduction in blood glucose level by improving urinary glucose excretion, which inhibits the stimulation of insulin secretion, leading to increased glucagon level and decreased insulin level. The lower insulin-to-glucose ratio increases liver gluconeogenesis and adipose tissue lipolysis, enabling the release of free fatty acids, which leads to ketogenesis in the liver (14).

### Fracture and Reduced Bone Mineral Density

The FDA has added the increased risk of fracture and decreased bone mineral density (BMD) as adverse effects on the canagliflozin label. Both animal studies and clinical trials have revealed that canagliflozin might affect bone microarchitecture, increase bone resorption, and reduce total hip BMD (83–85), which may be partly due to weight loss and reduced estradiol levels (84).

Studies on other types of SGLT2 inhibitors, such as ertugliflozin and dapagliflozin, did not identify their detrimental effects on bone turnover and BMD (86–88). However, meta-analyses and most clinical studies have revealed that the use of SGLT2 inhibitors, dapagliflozin, canagliflozin, and empagliflozin, was not significantly related to the increased risk of fracture (89, 90).

### Other Potential Adverse Effects

A decrease in estimated glomerular filtration rate (eGFR) level occurred during the initial treatment with canagliflozin; this decrease was weakened over time and was recognized to be unrelated to the severe renal adverse events (8). Other reported adverse effects of SGLT2 inhibitors include their volume depletion-related effects (such as postural dizziness, orthostatic hypotension, syncope, and reduced urine output) and osmotic diuresis-related effects (such as pollakiuria, nocturia, micturition frequency, and thirst).

## Discussion

In this review, we have summarized the findings of current studies on SGLT2 inhibitors. We have reported the advantages and adverse effects of SGLT2 inhibitors plus insulin therapy for the treatment of T2D (Table 2). SGLT2 inhibitors plus insulin therapy significantly improves glycemic control and reduce total daily insulin dose, body weight, and the risks of cardiovascular disease. Although the potential mechanisms for these effects were presented, several limitations still exist, thereby warranting the performance of further studies. As current studies only reported total daily insulin reduction with SGLT2 inhibitors plus insulin therapy, future studies should separately analyze the association between SGLT2 inhibitors and basal insulin or bolus insulin dose change. Furthermore, only few studies compared SGLT2 inhibitors with other antidiabetic drugs in conjunction with insulin therapy. As a result, more comparative studies should be carried out to elucidate the advantages and risks of these combination therapies. Finally, the mechanisms for the effects of the SGLT2 inhibitors were only partly revealed, thereby warranting a further study.

**Table 2:** The main benefits and risks of SGLT2 inhibitors plus insulin therapy.

**SGLT2 inhibitor** **+** **insulin therapy**	
**Advantages**	**Disadvantages**
1. Significant glycemia-lowering effect	1. Hypoglycemia (in the short term)
2. Reduction in daily insulin requirement	2. Genital and urinary tract infections
3. Weight loss	3. Diabetic ketoacidosis
4. Insulin sensitivity and β-cell function improvement	4. BMD reduction and fractures
5. Cardiovascular benefits 1) Reduction in cardiovascular mortality 2) Reduction in major adverse cardiovascular events 3) Decrease of blood pressur	5. Other side effects: Dizziness, orthostatic hypotension, syncope, pollakiuria, nocturia, micturition frequency, and thirst
6. Others possible benefits 1) Albuminuria reduction and improved albumin-to-creatinine ratio 2) Reductions in triglycerides and visceral adipose tissue volume 3) Decrease in IL-6	

*SGLT2, sodium–glucose co-transporter 2; BMD, bone mineral density*.

## Conclusions

SGLT2 inhibitors combined with insulin might serve as a promising therapy for the treatment of T2D. SGLT2 inhibitors have several advantages, and when combined with insulin, a significant decrease in HbA1c and FBG levels is achieved, enabling a reduction in the daily administered with insulin dose. Additionally, these inhibitors reduce body weight via glycosuria-induced calorie loss and negative energy balance, thereby increasing fat and sugar utilization and β-oxidation of fatty acids. SGLT2 inhibitors polarize M2 macrophages, affect adipocyte-derived hormones, and promote the expression of different β-cell-related factors, consequently improving insulin resistance and β-cell function. Other potential advantages of these inhibitors include a reduction in blood pressure, cardiovascular events, and visceral adipose tissue volume. SGLT2 inhibitors exhibit better glycemic control and cause higher body weight loss, higher blood pressure reduction, and lower cardiovascular events than do DPP-4 inhibitors. However, these inhibitors exhibit similar insulin-reducing effects. Although SGLT2 inhibitors caused less HbA1c reduction and, controversially, body weight loss than did GLP-1 agonists, their reduction of blood pressure and heart failure was more significant than those of the GLP-1 agonists. Further, as SGLT2 inhibitors are administered orally, therapy compliance can be improved.

The main risk of treatment with SGLT2 inhibitors combined with insulin therapy is mild GTIs; however, a good response is achieved with therapy. No difference in the incidence of hypoglycemic events and UTI was noted by treatment with the inhibitors compared to placebo. As euglycemic diabetic ketoacidosis, bone adverse effects, and other adverse effects have been reported, further studies are warranted. According to the findings of recent studies, treatment with SGLT2 inhibitors plus insulin is a suitable regimen for patients with T2D, especially those requiring a high insulin dose daily and those with insulin resistance, obesity, or high risks of cardiovascular disease. Large-scale and long-term clinical studies are, however, needed to confirm such findings.

## Author Contributions

All authors contributed equally to the writing, revision, and editing of this manuscript.

### Conflict of Interest

The authors declare that the research was conducted in the absence of any commercial or financial relationships that could be construed as a potential conflict of interest.
